# Simplified Algorithm for Determining Value-Based Pricing of High-Tech Medical Devices in the Tuscany Region of Italy

**DOI:** 10.7759/cureus.86743

**Published:** 2025-06-25

**Authors:** Andrea Messori, Beatrice Tognarelli, Janette Monzillo, Sabrina Trippoli

**Affiliations:** 1 Regional Health Service, Health Technology Assessment (HTA) Unit, Regione Toscana, Firenze, ITA; 2 Pharmaceutical Service, Ente Servizi Tecnici Amministrativi Regionali (ESTAR) Toscana, Firenze, ITA

**Keywords:** cost-effectiveness, cost-utility, medical devices, value-based pricing, willingness-to-pay threshold

## Abstract

In Europe, value-based pricing (VBP) is widely used for pharmaceuticals but less frequently for medical devices. Medical device prices are generally set at the regional level and rarely at the national level. Therefore, a simple algorithm for estimating VBP can be useful.

In Tuscany (a region with 3.7 million inhabitants), prices for high-tech medical devices have been set by a regional organization since 2018. These prices represent a mandatory condition for purchasing these devices but are generally determined empirically. In this context, we present a simplified algorithm for VBP estimation, which we have applied retrospectively to devices approved in the first half of 2024. The algorithm is based on the primary clinical endpoint used in the device studies and on health-care costs. It represents a modified version of the previously described added therapeutic value (ATV) model. To analyze the results of our experience, we performed a correlation analysis between VBPs and real prices.

Our algorithm was applied to 17 devices. In 13 cases, the VBP could be estimated, while in the remaining four cases, the algorithm failed. In cases where the calculation was successful, the correlation between VBP and real prices was highly significant (R^2^=0.929; 95% confidence interval (CI), 0.7747 to 0.9789; p<0.001).

In our previous experiences between 2019 and 2023, we attempted to apply a complete economic analysis to 239 newly approved devices and found that such an analysis was unfeasible in 80% of the cases. This simplified algorithm can be useful to determine a preliminary estimate of VBP when a more complete economic analysis is unfeasible.

## Introduction

In Europe, value-based pricing (VBP) is widely used for pharmaceuticals [1-5], but only occasionally for high-tech medical devices [6-9]. There are many reasons for this difference. First, national negotiations of reimbursed prices for pharmaceuticals have become a systematic practice in most European countries, thanks to the existence of national drug agencies responsible for these price negotiations [1,3]. By contrast, prices for medical devices (especially high-tech devices) are rarely set at the national level, with the sole exception of France [10]. Most often, reimbursed prices for devices in Europe are set either at the regional level or even at the level of individual hospitals, mainly due to the absence of a national regulatory agency responsible for price negotiations. Another reason why VBP is used much more frequently for pharmaceuticals than for devices is related to the quantity and quality of clinical studies supporting pharmaceuticals versus devices. It is well known that drugs are generally supported by adequate clinical evidence, but this is not the case for many devices [6]. The most important difference relates to comparative trials, which are almost always available for National Health System (NHS)-reimbursed medicines but are generally not available for devices.

To avoid setting prices reimbursed by the NHS for devices in the absence of any objective criterion, an appropriate methodology must be applied systematically to any newly introduced high-tech device. Pharmacoeconomic models based on cost-effectiveness, cost-utility, and cost-benefit approaches have been published in scientific literature for both pharmaceuticals and devices. However, in the case of devices, these publications have two very serious limitations: first, the result in terms of estimated VBP is generally kept at the level of an academic exercise because the institutions that carry out this research are not the same ones that take responsibility for the procurement of devices for the NHS; second, the number of these publications covers a very small percentage of the total number of newly introduced devices [2], thus leaving many devices out of any pricing scheme; to explain this drawback, we have shown that basic clinical or economic information is often missing also for class II or III devices [6].

To allow VBP to be widely applied to newly introduced devices, a simple, versatile method is needed. In this context, while standard methods of Health Technology Assessment (HTA) are able to propose complex and efficient pricing models, these proposals, however, are unsuitable for a systematic application to all newly developed devices because the basic information needed for their application is frequently lacking; at best, they occasionally provide a complete report for a few specific devices in a purely theoretical context where the estimated VBP is not adopted for the procurement of devices for the NHS.

In methods aimed at determining the VBP, a simplified approach has been described by Prieto-Pinto et al. [11], which relies on the added therapeutic value (ATV), that is, the incremental clinical benefit determined by the new product compared with the standard of care; this implies to measure the therapeutic advantages of new products (and their economic advantages, if any) compared with the best alternative already available to the clinicians.

In the present article, we describe a local experience of VBP that has employed a modified version of the above-mentioned ATV model [11]. In our model, the therapeutic advantages of the new device versus the old one have been limited to three parameters: (a) the primary endpoint of the clinical trial; (b) the percent improvement (if any) in this clinical endpoint, which is determined by comparing the endpoint between the new device vs the old one; (c) the impact (if any) on the health-care expenditure per patient of the new device compared with the old one.

We present a retrospective pilot study of 17 high-tech devices that were approved by our regional HTA body in 2024. We compared the real price of each device with its VBP, which was determined using the aforementioned approach. We employed this simplified method due to the limited data available on medical devices compared to pharmacological products. Thus, our algorithm's novelty lies in its applicability when information on the device is limited; this is particularly useful in Italy, where there is a lack of national pricing frameworks for devices. This lack assigns the difficult task of determining prices to regional HTA bodies (or individual hospitals in regions without an HTA body). 

## Technical report

Methods

The Tuscany Context in the Management of High-Tech Devices

In Tuscany (an Italian region with 3.7 million inhabitants), the approval of new high-tech devices is managed by a regional committee of 22 experts (called Centro Operativo (CO)). For this purpose, high-tech devices are defined as those belonging to risk class IIb and III. To make a decision on the procurement of any new high-tech device, a mini-HTA report is firstly redacted by a subgroup of the CO (usually three to five members) and then discussed in the monthly meetings of the CO. When the mini-HTA report is approved by the CO, the document is published on the regional website of the CO (https://www.regione.toscana.it/-/prodotti-hta#schede), where its full text is freely available in two languages (Italian and English). In each document, the English version is reported at the end of the Italian version. For many years, the Tuscany region has, in fact, systematically published the HTA reports for all newly introduced devices of risk class IIb and III. These mini-HTA reports are intended to be an evidence-based reference on new devices available to all health-care professionals of our region. They are also a tool to support an appropriate use of the new devices that receive regional approval for their acquisition.

Description of the Simplified Pricing Algorithm

**Figure 1:**
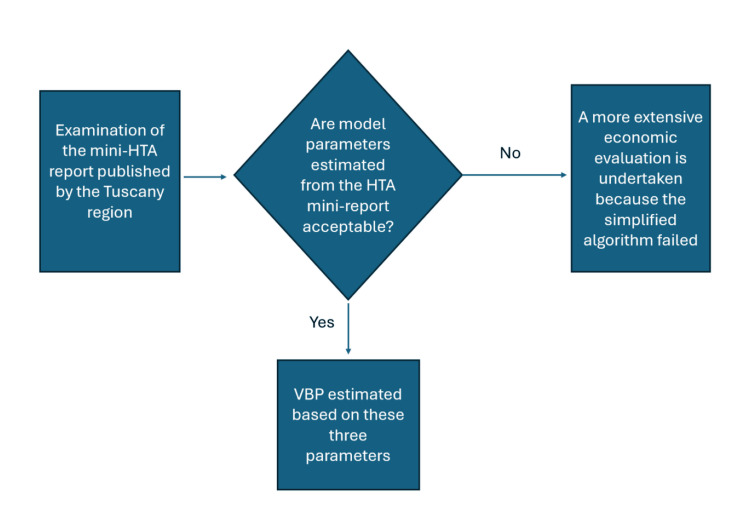
The decision process of our proposed simplified algorithm. Credit: Created by Sabrina Trippoli.

The algorithm for determining the VBP (Figure 1) resembles the ATV approach published by Prieto-Pinto et al. [11]. Our algorithm has been designed to be as simple as possible to enhance the degree of its applicability to new high-tech devices. The starting box of the decision algorithm is the “Examination of the mini-HTA report published by the Tuscany region” regarding the device under examination. The second step of the algorithm is the selection of an estimate for each of the three parameters of the ATV mode. These three parameters include: (a) the primary endpoint of the clinical study of the new device; (b) the percent improvement in the comparison of this endpoint between the new device vs the best available alternative (i.e. the comparator); (c) the difference in the economic impact (if any) between the two devices expressed in health-care cost per patient and without including the device price.

As a first option, these three parameters are determined by the authors of the mini-HTA report. The acceptability of these three parameters and of the consequent VBP is then discussed in the first monthly meeting of the CO where all its members are present. If the CO approves the HTA report and the respective VBP, the VBP is published in the internet website along with the mini-HTA report on the device under examination. If the decision is negative, the conclusion in terms of device procurement is that the simplified algorithm has failed so that a more complete economic analysis on the device is undertaken. In virtually all cases, parameters (a) and (b) are derived from the articles cited in the reference list of the report. Parameter (c) can either be estimated from our regional data or can be derived from published articles focused on cost or cost-effectiveness.

Since the experience described herein is a retrospective one, the values of VBP estimated through our pricing algorithm are presented in this article but cannot be found in the mini-HTA reports published on our regional website.

Description of the Regional Experience of the Tuscany Region

The present article describes the experience conducted by the CO on the consecutive requests for a new high-tech device received in the first semester of 2024. Our article limits the description of our experience to the application of the simplified pricing algorithm. Cases where the CO decided that a more complete economic analysis was needed are not described herein but will be the subject of a separate report.

Mathematical Details of the Model

This version of the ATV model implements the simplest form of a cost-benefit analysis, and so no willingness-to-pay threshold is adopted. The equation aimed at determining the VBP is as follows (where SOC=current standard of care):

$\text{Value-based price (VBP)} = (\text{Price of SOC}) \times \left[1 + \frac{\text{Percent improvement in clinical benefit}}{100} \right] + \Delta \text{Health-care cost}$

where

· “price of SOC” is expressed as €/patient;

· “improvement in clinical benefit” is expressed as a percentage; its sign is always positive with the exception of cases in which the device has worsened the clinical outcome;

· “(Δhealth-care cost”) is the increase or decrease in health-care cost per patient determined from the comparison of treatment group vs controls (or from the comparison of treated patients evaluated after treatment vs before treatment); in particular, its sign is positive if the new device has determined a saving and vice versa.

For each new device, the information represented by the VBP along with its three parameters is subjected to the evaluation of the CO in one of its monthly meetings; if the CO decides that the quality of the analysis is insufficient, the conclusion is that the algorithm has failed and a more complete cost-effectiveness analysis is undertaken. We did not assess the quality of the clinical trials from which these parameters were derived, mainly because the field of clinical trial quality assessment has not yet developed a method to convert the score generated by the assessment method into monetary units. In order to deal with this issue, we therefore made a simplified choice by assuming that clinical trials indexed in PubMed were the only type of clinical evidence suitable for inclusion in our analysis. Consequently, any clinical trials not indexed by PubMed were considered inappropriate for any of our analyses. We recognise that this is a very simplistic criterion, and we are aware that the limited information generally available for medical devices prevented us from setting a mandatory inclusion criterion of better quality, as this would have meant excluding more studies from our analysis.

One important exception to the model described above is when the mini-HTA report has identified, in the scientific literature, a complete cost-effectiveness analysis aimed at the estimation of the VBP of the new device in a European context (i.e. using the € as the currency of the analysis). In these cases, which are quite rare, the VBP estimated by the full economic analysis was used for the purposes of our study rather than the VBP value calculated by our simplified model.

Finally, in some cases, when a clinical study comparing the new device with the old one was not available but there is some evidence in support of the new device, the CO decided that the new device could be purchased provided that its price does not exceed the price of the old device; in such cases, the value of clinical improvement was set at 0%.

Statistical Analysis

Pearson’s correlation coefficient was determined to compare the VBP for each new device with its real price.

Devices Included in Our Analysis

We included in our analysis all devices of risk class IIb and III, that were proposed after January 1, 2024 for being purchased by our regional health system, thus becoming available to all public hospitals of our region. It should be kept in mind that more than 90% of the hospitals in our region belong to our public health-care system.

Results

Devices Included in Our Analysis and Estimation of VBP

The devices approved by our HTA centre after January 1, 2024 were 17. Their characteristics are summarised in Table 1. According to the decision made by our CO, our simplified pricing algorithm failed in four cases (coded 302, 294, 296 and 292) out of the overall series of 17 devices included in the analysis (23.5% failure rate).

**Table 1:** Characteristics of the 17 devices included in the analysis. ^†^The plus sign is used when the endpoint is improved by the new device while the minus sign indicates that the new device has determined a worsening of outcomes. ^††^A shorter procedural time by 21.68 min contributed to these savings for PFA. ^&^Real price. ^§^In this case, the procurement of this device was not approved. *In these cases, a complete economic analysis focused on the device was undertaken. **This clinical benefit was assumed to be 0.05 QALYs, which translated into €3000 according to a willingness-to-pay threshold of €60,000/QALY; this threshold has been formally recognised by the Tuscany Region for the purpose of the evaluation of innovative devices [24]. Abbreviations: VBP: Value-based pricing; NA, not applicable; PFA, pulsed-field ablation; CRYO, cryoballoon ablation; QALY: quality-adjusted life year.

Device (with identification code)	Device price^&^ (€)	Comparator	Comparator price (€)	Clinical endpoint	Percent relative improvement in the endpoint^†^	Documented savings in health-care expenditure (€)	Value-based price (€)	Applicability of the pricing algorithm	Reference to clinical outcomes
Flow Triever (No. 276)	8000	Systemic thrombolysis	809	Composite endpoint of multiple causes of unsuccess (e.g. hypotension)	+73.4% (17% vs 63.9)	0	1403	Yes	[12]
Hot Spaxus (No. 303)	5000	Hot Axios	6000	Severe bleeding (i.e. bleeding requiring transfusion and/or intervention)	+77.9% (1.5% vs. 6.8)	0	10674	Yes	[13]
Endovascular system Ekosonic (No. 126)^§^	3360	Catheter-directed thrombolysis	363	Reduction of systolic pressure in pulmonary artery	0%	0	363	Yes	[14]
Circular suturing machine for open surgery (No. 307)	400	Conventional circular staplers	306	Anastomotic bleeding	+87.3%% (0.7% vs 5.5%)	0	573.14	Yes	[15]
Farawave (No. 309)	5500	Thermal ablation	3590 (calculated from 3090 for radiofrequency ablation and 4090 for cryoballoon ablation); Value included in VBP calculation=€3590	No improvement in clinical outcomes	0%	Savings of PFA (excluding kit costs) vs CRYO=€850 per patient; vs RFA=1,301 per patient. Value included in VBP calculation=€1075^††^	4665	Yes	[16,17]
Intellis Surescan MRI (No. 301)	22600	Traditional spinal cord stimulation	14640	Low back pain responder rate	+56% (80.1% vs 51.2%)	0	22800	Yes	[18]
Urolift (No. 289)	1575 (an average of 3 devices per patient)	Transurethral resection of the prostate (TURP)	1351	BPH6 endpoint recovery (a composite of six elements)	+57.6% (82% vs 53%)	0	2129	Yes	[19]
Fitostimoline Plus (No. 302)	29.6	Advanced dressings with antiseptic properties are used, followed by hyaluronate gauze	30	NA				Failed algorithm due to lack of comparative data*	-
Signia small (No. 294)	1500	Sutures with larger diameter vascular loaders	-	NA				Failed algorithm due to lack of comparative data*	-
Tricvalve (No. 296)	24000	Not selected	-	NA				Failed algorithm due to lack of data regarding an adequate comparator*	[20]
Venaseal (No. 297) for cyanoacrylate closure (CAC)	620	Radiofrequency intervention (Catheters Closure Fast for venous radiofrequency ablation (CFT7-100 and CF7-360 by Covidien)	392	Freedom from recanalisation at 60 mos (CAC vs radiofrequency ablation)	+7.3% (91.4% vs 85.2%)	0	420.616	Yes	[21]
Cardioband Tricuspid (No. 287) used for transcatheter annuloplasty	22000	Trans- catheter edge-to-edge repair with TriClip®, PASCAL or off-label MitraClip	Pascal or Triclip, or off-label Mitraclip, all priced at €22,000	No difference in outcomes	0%	0	22000	Yes	[22]
Motiva Ergonomix (No. 293)	950 (determined from a range of 600 to 1300)	Smooth round silicone gel breast implants	472.5 (determined from a range of 315 to 630)	Expected reduction in the inflammatory response	0%	0	472.5 (i.e. the same price as the device currently employed)	Yes	No comparative study
Cardiomems (No. 305)	12000	No remote monitoring of the patient	0	QALYs	Gain of 0.1908 QALYs per patient	Savings of €1733 in hospitalizations	8475	A complete cost effectiveness study is reported in the mini-HTA report [25]	
Optilume (No. 310)	2000	Uteroplasty surgery	3059	Success of the intervention	0%	0	3059	Yes	-
Fixnip (No.292)	900	No other such device available	0	Reconstruction of the areola-nipple complex	0%			Failure of the algorithm due to the lack of data for valuing this benefit	-
Neovasc Reducer (No. 315)	6350	Sham procedure	0	Quality of life	Improvement at 6 months in quality of life of 10 points on a 100-point scale (from 7.6 points to 17.6 points)**		3000		[23]

Table 1 reports both the real price for each of these 17 devices compared and the estimated values of VBP; furthermore, Table 1 shows the detailed references reporting the specific clinical information (consisting of the above-mentioned three parameters), which was employed for model-based estimations of VBP.

**Figure 2:**
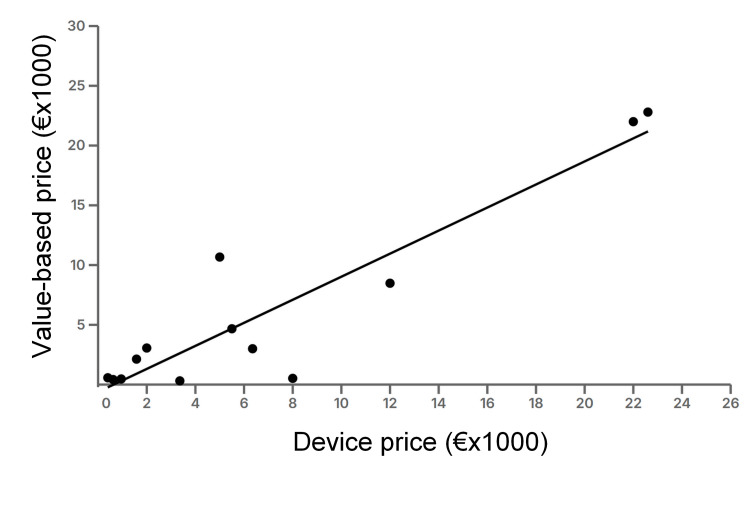
Value-based price vs real price for 13 high-tech devices: correlation and linear regression analysis. The correlation coefficient is highly significant (R^2^=0.929; 95% confidence interval, 0.7747 to 0.9789; p<0.001); the regression line is the following: y=0.9653; x=-552.9477. Image credit: Created by Andrea Messori.

Finally, Figure 2 shows the correlation graph between VBPs and real prices for the 13 devices for which the algorithm was successful; the correlation was highly significant (R^2^=0.929; 95% confidence interval, 0.7747 to 0.9789; p<0.001). Two outliers stand out in Figure 2: the first is Hot Spaxus, which has a real price of €5000 but an estimated VBP of €10,674; the second is Flow Triever, which has a real price of €8000 but an estimated VBP of only €1403.

## Discussion

First of all, the main limitation of the present study must be stressed: the ATV model for VBP estimation is clearly a rudimentary tool and has a lower methodological quality than a complete cost-effectiveness analysis focused on the medical device. Nevertheless, the proposal described herein is supported by several important reasons:

1. All attempts to systematically identify an adequate cost-effectiveness or cost-benefit model for a consecutive series of new high-tech devices have failed thus far without exception, demonstrating that such an analysis is only available for at most 30% of the new devices [2];

2. In the absence of a complete cost-effectiveness or cost-benefit analysis, the typical evaluation of the device price is currently based on fully arbitrary criteria, most frequently by consulting a decision committee (called “Collegio Tecnico” in Italy) who generally does not perform a systematic evaluation of the clinical evidence; for example, in the widely used method of 30% for price vs. 70% for safety/efficacy, the score up to 70 points assigned to efficacy can be indifferently as low as 10 points or as high as 60 points depending on the committee that has been consulted; there is currently no rule on this issue, but nevertheless, this approach continues to be used very widely. With regard to the name of the pricing method described in this paper, although we are aware that our method is a minor variation of the best alternative method, we have given it the name "Tuscany Value-based Pricing Method" in order to facilitate the citation of analyses based on this approach.

The main advantage of the ATV model is that a common pathway is proposed to determine the initial estimate of a hopefully appropriate price for all new high-tech devices. While the precision of the ATV model may be questioned, nevertheless its level of methodological quality is clearly better than the current practice based on the use of no specific criteria or objective models. Of course, the ATV model should not be used when a high-quality cost-effectiveness analysis is available, but we know that this happens in a minority of cases.

In summary, when assessing a new device using our VBP algorithm, we emphasise that the decision on whether or not to purchase the device depends to a large extent on what price the manufacturer has asked for. On the one hand, interpreting the clinical evidence supporting the new device is a fairly straightforward task, mainly because this interpretation can be based on well-established and well-known rules. On the other hand, deciding on an acceptable price for the new device is more challenging because the construction of the VBP according to the principles of cost-effectiveness is hampered by many obstacles, among which the lack of essential pharmacoeconomic information (with specific reference to the country considered in the analysis) plays a major role. In these circumstances, the availability of an acceptable estimate of the VBP of the device is extremely useful. In fact, with regard to the decision process leading to the approval or not of the purchase of the device, our rules suggest that the first step is to make an estimate of the device's VBP; then, when available, this estimate is managed as a useful piece of information in the context of all the factors influencing the final decision to purchase the device; however, there is no mandatory rule regarding the interpretation of the VBP so that the CO's final decision is made as an expert-based decision. Therefore, the expert-based opinion takes precedence over the price level of the new device generated by our simplified VBP algorithm. In 2025, we have continued with further experience based on the "Tuscany value-based pricing" method, the results of which are encouraging [25].

## Conclusions

Based on our experience between 2019 and 2024, we attempted to conduct a comprehensive economic analysis of a large number of newly approved high-tech devices, but found that this was unfeasible in 80% of cases. This simplified algorithm can therefore be useful for providing a preliminary VBP estimate when a more complete economic analysis is not possible.

In conclusion, this proposal is important because it addresses an unmet need in device pricing. To our knowledge, no empirical pricing algorithm has been developed yet for high-tech devices. This means that prices across Europe are often set arbitrarily, in the absence of a specific method. Therefore, our empirical VBP method can represent a practical advancement in this complex area.
